# Dog Owners’ Perspectives on Canine Heart Disease in Klang Valley, Malaysia

**DOI:** 10.3390/ani12080985

**Published:** 2022-04-11

**Authors:** Norhidayah Noordin, Kuan Hua Khor, Kuan Siew Khor, Yi Jun Lim, Yong Chong Lee

**Affiliations:** 1 Faculty of Veterinary Medicine, Universiti Malaysia Kelantan, City Campus Pengkalan Chepa, Kota Bharu 16100, Kelantan, Malaysia; norhidayahnoordin94@gmail.com; 2 Department of Veterinary Clinical Studies, Faculty of Veterinary Medicine, Universiti Putra Malaysia, UPM, Serdang 43400, Selangor, Malaysia; vonlajune6@gmail.com (Y.J.L.); wayne_lee25@msn.com (Y.C.L.); 3 Department of Management, Sunway University Business School, Petaling Jaya 47500, Selangor, Malaysia; kuansiewk@sunway.edu.my

**Keywords:** awareness, knowledge, canine heart disease, theory of planned behavior, perceived behavioral control, empathy

## Abstract

**Simple Summary:**

Heart disease is a common chronic illness among dogs that often requires long-term treatment. This study investigated owners’ awareness and knowledge about canine heart disease, followed by their intention to treat with respect to the Theory of Planned Behavior. The attitude, subjective norm, and perceived behavioral control (PBC) in predicting dog owners’ intention to treat canine heart disease, with empathy as moderator, was analyzed. The majority of dog owners have a high awareness of the disease denoted by their ability to identify 5 to 8 out of 12 common clinical signs. Although seeking treatment is a common effort by dog owners, the cost was the barrier that curtailed their intention. If dog owners improve their attitudes, receive support from spouses and other family members, and increase their confidence in managing their pets, this can increase the pursuance of heart disease treatment. Owners with low empathic concern can be motivated to treat by cultivating perceived behavioral control. Therefore, continuous education may improve owners’ preconceived ability to provide care, and veterinarians may play an essential role in encouraging treatment in dogs diagnosed with heart disease.

**Abstract:**

Canine heart disease often requires long-term treatment, which involves a continuous commitment from the dog owners. In addition to investigating their awareness and knowledge, the Theory of Planned Behavior was applied to also analyze attitude, subjective norm, and perceived behavioral control (PBC) of the dog owners, with empathic concern as a moderator in predicting intention to treat canine heart disease. Through a convenience sampling approach, 261 respondents, who were clients of University Veterinary Hospital, Universiti Putra Malaysia (UVH-UPM), with experience in owning or caring for dogs, were recruited. While the majority of the respondents (83.5%) claimed that they were aware of canine heart disease, most respondents (45.6%) could only identify 5 to 8 (Fair) out of 12 of the salient clinical signs. Most dog owners (92.3%) were willing to seek treatment if the pet dogs were affected, although the intent is deterred by cost (39.5%). In this study, attitude, subjective norms, and perceived behavioral control were significant predictors for the intention to treat. Dog owners with low empathic concern can be motivated to treat affected dogs by cultivating perceived behavioral control. Therefore, continual education may improve dog owners’ preconceived ability to provide care, and veterinarians may play an important role to encourage treatment in dogs diagnosed with heart disease.

## 1. Introduction

Heart disease is commonly diagnosed among dogs; it accounts for approximately 10% of dog cases presented in primary veterinary healthcare in the United States [1]. However, in Malaysia, the scarcity of retrospective data on canine heart disease in Klang Valley yielded a prevalence rate of 6.30%, where myxomatous mitral valve disease (MMVD) was the leading condition [2]. In addition, the majority of the total MMVD patients diagnosed were presented with overt clinical signs of congestive heart failure [2]. MMVD is a condition where the mitral valves become thickened, elongated, or prolapsed, causing backflow of the blood into the atrium during systole [3]. In an earlier retrospective study, dilated cardiomyopathy where heart chambers lose their contractility [4] was ranked second (6.32%) to MMVD (64.2%), followed by heartworm disease (3.85%) [5].

Depending on the different types of heart disease, either with volume and/or pressure overload, perpetual neurohormonal activation and cardiac remodeling will ultimately lead to congestive heart failure with clinical signs such as coughing, syncope, and exercise intolerance, which may interfere with both owners’ and dogs’ quality of life (QoL). The ultimate aim of the lifelong treatment is to manage these clinical signs, prolonged life span and ensure good QoL, i.e., the ability to exercise better, cough less, eat well and maintain a good body condition score [1]. Due to the irreversible nature of the disease, lifelong medications have been shown to support heart function and reduce the intensity of clinical signs, prolonging the asymptomatic periods in both MMVD [6], and dilated cardiomyopathy patients [7]. Treatment for congestive heart failure in dogs includes a combination of medications such as diuretics, inodilators, and angiotensin-converting enzyme inhibitors along with supplements, and dietary managements such as low sodium diets are also important in supporting the patient long-term.

Pets are entirely dependent on their owners as caretakers, providers, and decision-makers in their daily life, and therefore, they play an important role in treatment and management if a medical condition arises [8]. Without a good level of awareness and knowledge about heart disease, owners may miss the chance for early detection and intervention. After obtaining a diagnosis, it is crucial that dog owners are committed to supporting long-term treatment. To our knowledge, the factors that influence their behavior in making decisions to treat dogs with heart disease have not been identified.

Various social psychological theories have been developed, and the Theory of Planned Behaviour (TPB) is considered robust in predicting behavior [9]. To date, one study looked at the intention of cat owners in treating heart disease cats [10] and found that TPB items were significant predictors of intention to treat. However, to our knowledge, no similar studies have been conducted to evaluate dog owners. TPB posited that behavior can be predicted primarily by intentions, which are guided by attitudes, subjective norms, and perceived behavior control [11]. Intention to perform a particular behavior is an antecedent of willful behavior, which remains a behavioral disposition until an opportunity to act manifests. Attitude is defined as the function of an individual’s belief in a behavior. Subjective norms refer to the perceived expectation of others or social pressure to engage in a particular behavior, whilst perceived behavioral control (PBC) refers to the degree to which the person believes that he or she can perform a given behavior without difficulty [12].

Due to the presence of bonds shared between humans and pets, pet owners may experience empathy upon witnessing relatable suffering in their pet dogs. A positive correlation between empathy and a positive attitude towards animals has been documented [13,14]. Individuals with high empathetic concern can share feelings with people in need and have a greater tendency to provide welfare to others to relieve hardship [15]. Therefore, it is interesting to investigate whether empathic concern originating from a long-term human–pet interaction may contribute towards the owner’s intention to treat canine heart disease should their dog be diagnosed with one. Within a theorized association between intention to treat and behavior, the absence or inconsistency of the behavior may be attributed to a third variable that moderates the direction and strength of the relationship [16]. Receiving a diagnosis may not necessarily predict the intention to treat. A revelation on the reality of the disease and management of the disease through consultation may moderate owners’ intention to treat and support pet dogs, especially if the pet dogs are loved as part of a family in the household. It can be speculated that the presence of empathic concern might strengthen or weaken the interaction between the components of TPB and the owners’ intention to treat canine heart disease.

This study assessed the awareness, knowledge, and willingness of dog owners to seek treatment for canine heart disease. Factors affecting intention to treat among dog owners and the role of empathy were also identified using an extended model of TPB with empathic concern as moderator. The hypotheses of this study were as follows; (i) dog owners’ awareness and knowledge on canine heart disease are low, and (ii) attitude, perceived behavioral control, and subjective norms positively affect intention to treat canine heart disease, and empathy moderates these positive relationships. Information obtained from this study and the factors identified can be applied by veterinarians to strategize and improve the veterinary healthcare systems besides providing provisional support to dog owners.

## 2. Materials and Methods

### 2.1. Respondents

Convenience sampling was used, and the study took place at University Veterinary Hospital, Universiti Putra Malaysia (UVH-UPM), a primary veterinary healthcare facility. The inclusion criteria of targeted respondents were: (i) owners or carers of pet dogs at UVH-UPM, (ii) Malaysians who were ≥18 years of age, and (iii) owners who were responsible for the healthcare management of their pet dog. All respondents were approached by the same researcher (NN), while they were in the waiting lounge. Potential participants were carefully selected to avoid unnecessary stress to the owners and the dogs. The researcher only approached owners with calm dogs and owners with a non-emergency appointment. Owners that came with partners were preferable as the partner can help monitor the dog while the owner can complete the questionnaire without much distraction. To avoid social desirability bias and ethical implications, no staff or clinicians were involved in approaching the respondents. The researcher introduced herself as a postgraduate student instead of a veterinarian. The investigator assisted in translation or clarifications but refrained from giving opinions. The respondents were briefed and given a consent form to sign when they agreed to participate. The consent form included a brief explanation of the objectives of the research and confidentiality assurance that the information provided will be used strictly for the purpose of this study. The data collection took place from July to December 2020 (6 months). Precautions that were taken to reduce potential COVID-19 spread, included double-masking practice, communicating with potential respondents 1 m apart, encouraging the use of respondents’ pens, and frequent personal sanitization practices.

### 2.2. Questionnaire

The questionnaire was constructed in English. To improve the clarity of the wording used, a pilot study was conducted to identify difficult words and any misunderstanding of sentences and double-barreled questions among the measurement items. Feedback from the pool of respondents (*n* = 20, who consist of dog owners of different age groups and levels of education) was important to ensure that the questions were easy to understand prior to the actual data collection. A self-administered pen and paper survey was provided, with approximately 15 min allocated for answering. However, to abide by social distancing measures due to COVID-19, a digital format using Google Forms was created, which the respondents accessed through a QR code. Assistance was provided by the investigator (NN) on any queries raised by the respondents without providing any comments to avoid biases. As a token of appreciation, a multipurpose water-activated travel towel for owners (a non-sponsored item) was given upon completion of the questionnaire.

The first sections of the questionnaire collected information such as the demographic of the dog owners (age, gender, and monthly household income), length of experience (years) in caring for dogs, the purpose of having a dog in the household, level of awareness, and knowledge of canine heart disease, as well as barriers to seeking long-term treatment. A ten-point Likert-type scale was used for the respondents to judge their level of understanding of canine heart disease, where “Poor” understanding was 1–4, “Fair” was 5–7, and “Good” was 8–10. Respondents were required to go through a list of clinical signs by ticking “Yes”, “No”, or “Maybe”. Out of 24 clinical signs listed, 12 clinical signs related to heart disease described in layman terms were as follows: difficulty in breathing, panting all the time, panting even at rest, panting longer after a walk, fainting, seizure, poor body score (thin), exercise intolerance, coughing with phlegm, sleeping most of the time, looks lethargic, and swollen legs. The response “Yes” means that respondents were confident about the clinical signs related to heart disease, whereas the opposite was true for “No”. “Maybe” could be selected as the choice if owners were not sure about those clinical signs. For analysis, “Maybe” responses were automatically grouped as “No”. The respondents were graded “Poor” for identifying 0–4 clinical signs, “Fair” for 5–8 signs, and “Good” for 9–12 signs.

In the second section of the questionnaire, the respondents were prompted to rate agreement towards the multidimensional items proposed by TPB theory using a seven-point Likert-type scale as follows: 1: disagree; 2: somewhat disagree; 3: strongly disagree; 4: unsure; 5: agree; 6: somewhat agree, and 7: strongly agree. The questions applied TPB theory, where attitude, PBC, and subjective norm were examined in predicting the intention to treat canine heart disease among dog owners of both affected and non-affected pet dogs in a veterinary hospital (Appendix A). With regard to the intention to treat, owners were examined on their intention to perform practices related to treating and improving the well-being of pet dogs, such as treatment follow-ups, practicing suitable diet management, and administering prescribed medications. In the attitude section, owners were asked to rate their perceptions relating to heart disease management, which were regular check-ups, ensuring good health, beginning long-term therapy, and updating veterinarians on the well-being of the dog. For PBC, owners were asked to rate their status of supporting the long-term treatment, such as resources (time and money), nursing skills and commitment, and skills in administering medications. In the subjective norm section, the owners rated the importance of support from their family (parents/children), close friend(s), peers/colleagues, and spouse in seeking treatment for the pet dog. Finally, the owners were asked three questions to gauge their empathic concern, which were about how they would rate their tender and concerned feelings for animals, whether the pet dog was their family member, and whether they would describe themselves as soft-hearted person [17].

### 2.3. Statistical Analysis

Descriptive analysis, reliability, and normality of measurement items were analyzed using IBM^®^ SPSS^®^ Statistics Version 26. The Kolmogorov–Smirnoff test revealed that the data were not normally distributed and therefore were presented as medians. Kruskal–Wallis test followed by pairwise post hoc Dunn test with Bonferroni adjustments was used to investigate the differences between age, income, years of experience in taking care of pet dogs, and ability to identify clinical signs of heart disease on intention to treat [18]. Mann–Whitney U test was performed to compare the differences between gender on intention to treat [18]. Statistical significance was determined at a *p*-value of ≤ 0.05.

Subsequent analyses focused on applying variance-based structural equation modeling using SmartPLS Version 3.3.3 software to predict the intention to treat dogs with heart disease. Chin et al. (2019) [19] and Hair et al. (2020) [20] were used as a standard reference for reporting. Since both independent and dependent variable data were obtained from a single source, the common method variable (CMV) issue was addressed by testing full collinearity [21]. All variables were regressed against a common random variable and were considered to have no bias if the variance inflation factor (VIF) ≤ 3.3. Once again, the goodness of the measurement model was assessed based on convergent validity, where average variance extracted (AVE) should be greater than 0.5 and composite reliability (CR) should be greater than 0.7 [20]. Cronbach’s alpha value above 0.6 is generally considered acceptable. On the other hand, discriminant validity was established when the intra-construct item correlation was greater than the inter-construct item correlations, thus satisfying the Fornell and Larcker criterion [22]. Next, the variance inflation factor (VIF) was used to analyze collinearity between the formative indicators, where VIF <5 indicates the absence of multicollinearity issues. Finally, the significance and relevance of the formative indicators were assessed using the bootstrapping procedure, where indicators are retained due to (i) significance of outer weights and (ii) insignificance of outer weights but outer loadings above 0.5 [20].

**Figure 1:**
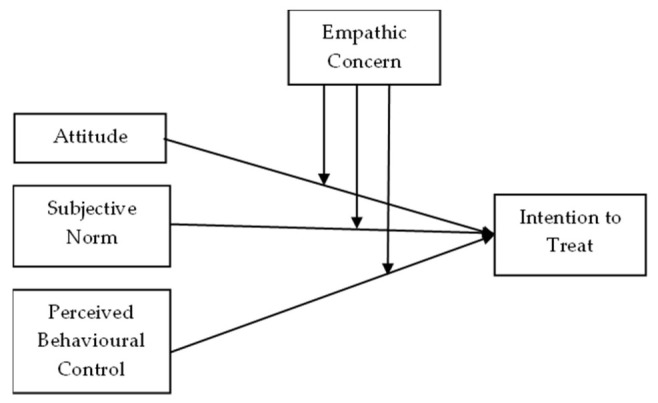
The research framework of the study exhibiting an extended theory of planned behaviour with empathic concern as the moderating variable.

Next, the structural path was assessed using the bootstrapping procedure with 10,000 sub-samples to treat non-normal multivariate data [23]. Apart from examining the influence of attitude, subjective norm, and PBC as predictors of intention to treat, this study also examined the moderating effect of empathic concern on the relationships mentioned above (Figure 1). Consequently, plots of significant interactions between the predictors and empathic concern [24], also known as the post hoc graph, were developed to illustrate the relationships in the circumstances of owners’ inherent level (low or high) of empathic concern.

## 3. Results

### 3.1. Descriptive Analysis

Only three dog owners declined to participate when approached, and a total of 261 respondents were recruited in this study. In the structural equation modeling, three respondents were excluded due to incomplete information given in that section. Based on observation, the majority of respondents attempted the questionnaire within 15 min, and it did not affect their waiting time at the lounge. There were 193 (73.9%) respondents who used the “pen and paper” questionnaire, while 68 respondents (26.0%) filled in the Google Forms.

Table 1 shows the demographic profile of the respondents: the majority were female (60.0%), more than 50 years old (29.5%), and with a monthly household income ranging from MYR (MYR 1 equals USD 0.24 as of 20 February 2022) 2001 to 5000 (32.0%). 

Table 2 reveals that the main purpose of respondents in keeping a dog was as pets for themselves (35.1%). In addition, a large proportion of the respondents (35.2%) have had dogs as pets for between 10.0 to 19.9 years. 

Table 3 reveals that the source of information sought by owners were mainly online sources (32.2%) followed by advice from veterinarians (30.7%) and pet books/newspapers/magazines (28.3%).

Table 4 showed that cost was the most common barrier for owners in seeking long-term heart disease treatment (39.5%). To the question of whether lifelong treatment is considered troublesome for the respondents, 28.5% of them answered “Yes”, while another 36.4% indicated “Maybe”. However, a majority (92.3%) agreed that they would be willing to seek treatment if the pet dog was diagnosed with heart disease.

Table 5 denotes that most of the dog owners (83.5%) were aware of the susceptibility of dogs to heart disease. When the respondents were asked to appraise their understanding of canine heart disease, only 7.7% rated themselves “Good”, 53.6% rated themselves “Fair”, and 38.7% acknowledged having a “Poor” understanding of the disease. Only 56.7% of the respondents could identify more than five clinical signs related to heart disease (Table 5).

**Figure 2:**
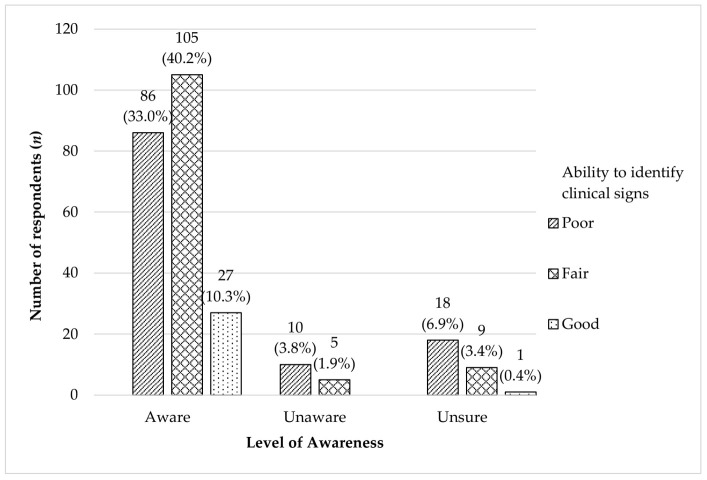
The relationship between the ability to identify clinical signs and the level of awareness of canine heart disease among dog owners (*n* = 261) in the study.

Figure 2 demonstrated that when comparing awareness against the level of knowledge on canine heart disease, many of the respondents who were aware of the disease were able to identify “Fair” numbers of clinical signs (5–8), and only 10.3% of them could recognize more than 8 clinical signs. Among the 12 clinical signs, most of the respondents had correctly identified difficulty in breathing (69.3%) as commonly observed, followed by lethargy (63.3%) and panting even at rest (49.8%).

**Figure 3:**
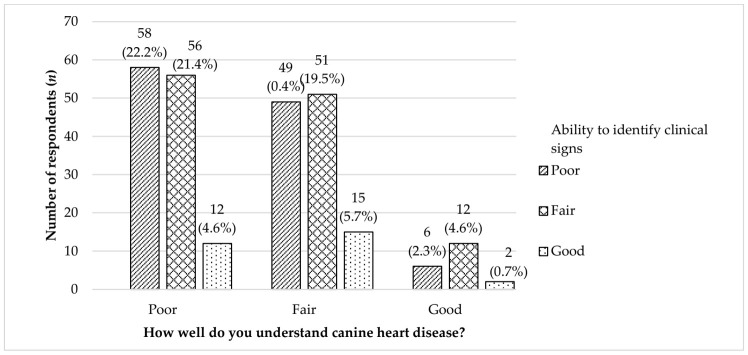
The relationship between the ability to identify clinical signs and the level of understanding on canine heart disease among dog owners (*n* = 261) in the study.

About 22.2% of the respondents who claimed to have a poor understanding indeed scored “Poor” in identifying clinical signs. Among the respondents that appraised their understanding as “Good”, the majority (12 out of 29 dog owners) could only identify 0–4 clinical signs (*n* = 6) or 5–8 clinical signs (*n* = 12) (Figure 3).

No significant differences (*p* > 0.05) were observed between age, income, and years of experience in having a pet. However, an association (*p* < 0.05) existed between different levels of scores for the ability to identify clinical signs towards the intention to treat canine heart disease. Respondents that scored “Good” had the higher intention (*p* < 0.05) to treat (Median, Md = 6.8) compared to those that scored “Poor” (Md = 6.4) (Table 1). Female dog owners had a higher intention to treat (*p* < 0.05) (Md = 6.6) compared to male owners.

### 3.2. Common Method Variable (CMV)

Table 6 showed that VIF obtained for all variables was ≤3.3 after both independent and dependent variables were regressed against a common random variable. Therefore, data did not run into CMV issues, and measurement model assessment was proceeded.

### 3.3. Measurement Model Assessment

Convergent validity was established because the AVE for all of the constructs was above 0.5 and Cronbach’s Alpha was between 0.65 to 0.79 (Table 7) Discriminant validity was established as composite reliability of all items being above 0.80 (Table 8). The square root of the variance was shared between each construct, and its items were greater than the intercorrelation of each construct and any other construct in the model, indicating that discriminant validity was achieved. These findings indicate that all measured items were suitable representatives of the respective variables.

### 3.4. Structural Model Assessment

**Figure 4:**
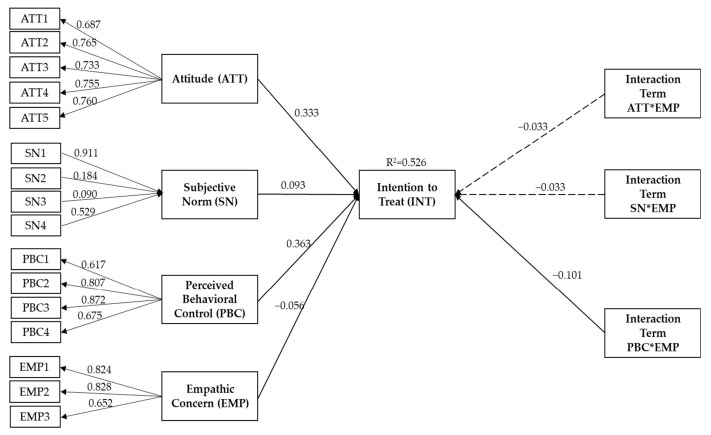
The output of structural model analysis showed the relationship between attitude, subjective norm, perceived behavioral control, empathic concern, and the interaction terms with the intention to treat. Solid line denotes significant relationship (*p* < 0.05), whereas dotted line denotes insignificant relationship. * Denotes the interaction.

The structural model assessment began with hypothesis testing, i.e., testing of the significance of the relationship between attitude, PBC, subjective norm, and empathic concern, and the moderating effect of empathic concern on items of TPB. The overall explained variance (R^2^ value) of 0.526 indicated that attitude, PBC, subjective norm, and empathic concern explained 52.6% of the variance in intention to treat. The path coefficient of the hypothesized relationship was meaningful, as the t-value is above 1.65 (significance at *p* < 0.05). Therefore, all three direct paths to intention to treat, i.e., attitude, subjective norm and the PBC were significant in predicting intention to treat (Table 9). Only one of three interaction terms, specifically the product of PBC and EC, was significant in predicting intention to treat. A simplified figure illustrating these relationships is depicted in Figure 4.

Based on the hypothesis testing, dog owners with a positive attitude toward canine heart disease management would have a higher intention to treat. Similarly, support from spouses and other family members and the idea of having the ability to manage the affected pet may encourage the intention to treat. The intention to treat among owners with low empathic concern improved when owners perceived that they can perform specific behavior without difficulty.

## 4. Discussion

In the management of canine heart disease, the integral part is treatment and nursing care. Therefore, the owners’ awareness and understanding of the diseases with good compliance practices towards treatment plans are some of the key factors for a successful treatment and management of chronic illness [25]. In this study, the level of awareness among the owners was high, although the ability to identify the clinical signs was fair. The respondents could identify crucial signs such as difficulty breathing, lethargy, and panting even at rest, which are common clinical signs of heart failure in dogs [26,27]. As preclinical stages of heart disease may be asymptomatic, recognizing these clinical signs early by the dog owners is of utmost importance. Furthermore, the ability to identify the wide range of clinical signs becomes helpful when monitoring the dogs that are undergoing treatment for congestive heart failure apart from maintaining a good QoL. However, it can be speculated that the lack of confidence on whether the clinical signs indicate heart disease may hinder the owners to bring their dog for veterinary check-ups and may instead opt for a wait-and-see approach. In a study among owners whose dogs had advanced heart diseases, the majority were found to prioritize the dogs’ QoL. Likewise, these owners were highly concerned about the potential sufferings that their pets might endure [28]. Therefore, it can be speculated that if dog owners are familiar with the dire consequences of the disease, they may be more open to the option of early screening and treatment as they comprehend the risk and prognosis of the disease.

As for the source of information, most of the dog owners developed a general understanding of dogs with heart disease based on online sources, followed by veterinarians and printed articles. While explaining the disease, veterinarians may guide concerned owners to reputable online sources [29,30]. This will offset the dangers of misinformation received and may prevent self-taught decisions such as switching the dog’s prescription based on personal online research [31]. It can be speculated that owners with better knowledge and improved understanding may have a greater intention to treat. These owners are presumably more informed on how the disease may affect the pet dog and therefore may have increased motivation in improving their pets’ health condition [32]. In terms of demographic profiles, female owners had higher intention to treat and were assumed to have higher empathy towards animals [33,34,35] due to the nurturing and caregiving instinct [36].

The cost of seeking long-term treatment remains a major concern in veterinary healthcare worldwide [10,32,37]. A similar scenario may exist in the Klang Valley due to the rising cost of living and declining national household income level [38]. This situation may lead to owners resorting to their management of the pets’ heart disease. Klang Valley represents a large urban region within Malaysia. It would be interesting to compare the monthly spending allocation on healthcare for their dogs based in different areas, i.e., urban, town, or rural, or based on the socio-economics of dog owners in Malaysia.

As an effective strategy, veterinarians can advise a management plan suited for both the dogs’ requirements and the affordability for the owners. Heart disease is no doubt incurable; however, it is important to educate owners to understand that the treatment may improve the QoL of affected dogs. Positive outcomes were seen post-therapy [39] might motivate owners to pursue and continue treatment as their effort shows improvement in their dogs. Recently, the concept of pet insurance is receiving positive attention in Malaysia. Veterinary centers may promote this as a solution to alleviate the burden of treatment costs. In terms of affordability in treatment cost, the veterinarian may suggest alternative drugs to the therapeutic regime, prioritize important medications or which test to be carried out as the disease progresses during long-term monitoring.

The overall attitude determined by subjective values was associated with the behavior and the positive or negative valence of the anticipated outcome [12]. Therefore, owners who associate tasks involved in managing dogs with heart disease and the positive outcome may be more inclined to treat their pet dogs. Education and encouragement on good practices of owners such as periodical check-ups and updating the veterinarians may instill good perception and strengthen the intention to treat. In pet ownerships, the human–animal bond of the respective household may be strong as pet dogs are fondly regarded as family members and the tasks of nursing and monitoring may be delegated among household members [40]. In this study, views and encouragement from spouses and other family members (subjective norm) were important when seeking treatment for pet dogs, which is similar to the scenario observed in human medicine [41,42]. Therefore, the involvement of spouses and other family members during consultations may facilitate decision-making as all members of the family can obtain the same quality of information directly from the veterinarian.

In this study, PBC was found to moderately affect owners’ intention to treat canine heart disease. The psychological perception of succeeding in the management of the disease in pet dogs may influence the owner’s effort, preparation, and performance [11]. A low sense of control over the management of chronically ill patients predisposed caregivers to stress and anxiety [43]. In the act of improving the PBC, veterinarians can revise treatment plans by considering owners’ capability, guiding them in nursing [44] and demonstrating effective skills [45] in key tasks, i.e., pilling and monitoring breathing rate. Other factors such as concurrent illness management of the heart-diseased dogs or other dogs at home may affect dog owners’ adherence to medication and routines [31]. Allowing owners to discuss their frustrations and issues during consultation [46] may discourage caregiver burden and establish a good rapport that influences owners’ motivation to initiate or continue long-term treatment [47,48].

The relationship between PBC and intention to treat canine heart disease was stronger among owners who scored lower in empathic concerns, a finding comparable to another study that looked at cats with heart disease [10]. Among empathic people, increasing the welfare of a person in need was the ultimate goal of their prosocial behavior [49]. Due to owners’ inability to relate to the pet dogs’ pain, additional care that requires extra effort may seem daunting among dog owners with low empathic concern. Therefore, where the owners seemed to lack empathic concern (i.e., not seeing the pet dog as a family member, not having a close bond with the pet dog), actions that provide owners with a sense of control over the disease management such as simplifying the treatment plan, reinforcing dog owners’ skills in medicating, and nursing the pet dogs may contribute to owners’ intention to treat canine heart disease.

This study is limited by the use of convenience sampling, which may have affected the generalizability of findings, as the results were collected from owners who have visited UVH-UPM over a particular time frame and are considered to have a certain degree of consciousness of veterinary health. In addition, despite numerous precautionary steps taken, the role of UVH-UPM as a veterinary hospital made social desirability bias hard to eliminate. The majority of the data were collected amid the COVID-19 outbreak between June to August 2020. We used an online questionnaire on top of paper and pen questionnaire distribution to accommodate the nationwide movement restriction order. The effect of different questionnaire distribution methods may have introduced some bias into the results. Through the use of Google Forms, the owner could not estimate the length of the questionnaire and may have carefully answered instead of blindly filling in to save time. Misunderstandings of the instructions can also be avoided as online forms were designed with features that facilitate respondents’ answering. We could not dismiss the impact of the global disaster on owners’ financial and psychological health, which may have affected their priorities towards their pet dogs. The findings from the TPB section in this study may not be reflective of dog owners who had or had not treated dogs with heart disease concerning their level of experience and knowledge, which is a good area to investigate in the future.

## 5. Conclusions

Although the majority of dog owners were aware of heart disease in dogs, their ability to identify hallmark signs of heart disease were fair. Online sources were favored most by dog owners when seeking information to better understand the disease. The cost was highlighted as the main barrier in supporting the long-term management of dogs with heart disease, but if owners were aware of the benefits of treatment, this might motivate them to continuously support their pet dogs. Attitude, subjective norms, and PBC positively influenced the intention to treat. Owners with a low empathic concern may have increased intention to treat if the PBC were strengthened. In this study, TPB items only account for half of the overall explained variance of intention to treat. More exploration in the field of study can be furthered to understand the owner’s intention to treat particularly in canine heart diseases. Providing resources and continuous education by a veterinarian may play an important role in boosting dog owners’ confidence and motivation in initiating treatment, especially in owners with low empathic concern. For owners with weak emotional bonds with their pet dogs, veterinarians can strategize and identify the difficulties that dog owners faced and motivate them to improve their intention to treat.

## Figures and Tables

**Table 1:** Demographic profiles of the participating dog owners (*n* = 261) and the association between owners’ gender, age, income level, experience in having pet dogs (years), and ability to identify clinical signs with intention to treat canine heart disease.

Demographic Profiles		Number of Respondents (*n*)	Percentage (%)	Association Tested with the Intention to Treat				
				Mann–Whitney U Test				
				Median	U	Z	*p*-Value	
Gender	Male	102	39.1	6.20	5552.00	−4.12	<0.001 *	
	Female	159	60.9	6.60				
				Kruskal–Wallis Test				
				Median	H	df	*p*-Value	Adjusted *p*-Value ^a^
Age (years old)	<20	10	3.8	6.40	2.03	4	0.73	-
	20–29	66	25.3	6.60				
	30–39	64	24.5	6.40				
	40–49	44	16.9	6.40				
	>50	77	29.5	6.40				
Monthly household income (Ringgit Malaysia, MYR)	≤2000	43	16.5	6.40	1.46	4	0.83	-
	2001–5000	86	33.0	6.60				
	5001–10,000	57	21.8	6.40				
	10,000–20,000	44	16.9	6.50				
	>20,000	31	11.9	6.60				
Experience in having pet dogs (years)	≤5.9	56	21.5	6.40	0.98	3	0.80	-
	6–9.9	77	29.5	6.40				
	10–19.9	92	35.2	6.60				
	>20	36	13.8	6.40				
Ability to identify clinical signs of heart disease	Poor (0–4)	114	43.7	6.40	7.20	2	0.027 *	0.026 *
	Fair (5–8)	119	45.6	6.60				
	Good (9–12)	28	10.7	6.80				

* *p* < 0.05; H, Chi-square test statistic; df, Degree of freedom; ^a,^ Bonferroni adjusted alpha level; U, Mann–Whitney U value; Z, Standardized test value.

**Table 2:** Purpose of having pet dogs among dog owners (*n* = 261).

Purpose of Having Pet Dogs *	Frequency of Selection (*n*)	Percentage (%)
As a pet for myself	175	35.1
As a pet for my children	42	8.4
As a guard dog for my property	74	14.9
As a companion	152	30.5
As a helper/guide for my disability	7	1.4
I am a breeder	3	0.6
I rescue dogs	42	8.4
Others	3	0.6

* Respondents were allowed to select one or more options.

**Table 3:** Source of information used by the dog owners (*n* = 261) in acquiring information on canine heart disease.

Source of Information *	Frequency of Selection (*n*)	Percentage (%)
Online sources	84	32.2
Veterinarian	80	30.7
Read up from pet book/magazine/newspaper	66	28.3
My relative or friends told me	58	22.2
I saw a poster available in the veterinary clinic	55	21.1
From a pet show/expo	49	18.8
From previous experience of having dogs with heart disease	48	18.4
I was given a pamphlet obtained from the veterinary clinic	23	8.8
Others	9	3.4
Total	472	100.0

* Respondents were allowed to select one or more options.

**Table 4:** Willingness to seek treatment, barriers in seeking treatment, and perception towards lifelong treatment among the dog owners (*n* = 261).

Items		Number of Respondents (*n*)	Percentage (%)
Willingness in seeking treatment if pet dog is diagnosed with heart disease.	Yes No Maybe	241 7 13	92.3 5.0 2.7
Barriers in seeking treatment * (* Multiple choice answers)	Cost-related Time-related No cure Others	103 70 79 9	39.5 26.8 30.3 3.4
Would lifelong treatment be troublesome?	Yes No Maybe	74 92 95	28.4 35.2 36.4

**Table 5:** Awareness, understanding of dog owners (*n* = 261), and their ability to identify clinical signs of canine heart disease.

Items		Number of Respondents (*n*)	Percentage (%)
Are you aware that dogs can suffer from heart disease?	Yes No Maybe	218 15 28	83.5 5.7 10.7
How well do you understand canine heart disease?	Poor Fair Good	101 140 20	38.7 53.6 7.7
Scores on the ability to identify clinical signs	Poor (0–4) Fair (5–8) Good (9–12)	113 119 29	43.3 45.6 11.1

**Table 6:** Full collinearity testing output.

ATT	EC	INT	SN	PBC
1.87	1.54	2.01	1.06	2.07

ATT: attitude, EC: empathic concern, INT: intention to treat, SN: subjective norm, PBC: perceived behavioral control.

**Table 7:** Convergent validity of the measurement items.

Items			Mean (SD)	Loadings ^1^/Weights ^2^	Cronbach’s α/CR	AVE
Attitude (ATT)	ATT1	It is wise to bring my dogs for his/her regular check-ups.	6.48 (1.00)	0.687	0.794/0.858	0.548
	ATT2	It is a good idea to ensure that my dog is healthy.	6.75 (0.68)	0.765		
	ATT3	It is wise to begin the life-long therapy (medication) for the benefit of my dog’s life.	6.45 (0.92)	0.733		
	ATT4	I will update my veterinarian during each check-up on my dog’s condition during treatment at home.	6.61 (0.77)	0.755		
	ATT5	I will get in touch immediately with my veterinarian when my dog suddenly looks sick.	6.64 (0.78)	0.760		
Empathic Concern (EMP)	EMP1	I often have tender and concerned feelings for animals.	6.48 (0.90)	0.824	0.664/0.814	0.597
	EMP2	My dog is a family member.	6.77 (0.62)	0.828		
	EMP3	I would describe myself as a pretty soft-hearted person.	6.17 (1.13)	0.652		
Intention to Treat ^3^ (INT)	INT1	I intend to follow up with my dog’s heart treatments.	6.51 (0.93)	0.756	0.691/0.811	0.523
	INT2	I intend to practice a low salt diet.	6.30 (1.28)	0.541		
	INT4	I intend to stop giving commercial treat(s) to my dog.	6.66 (0.74)	0.812		
	INT5	I intend to administer medications recommended by the veterinarian.	6.53 (0.98)	0.752		
Perceived Behavioral Control (PBC)	PBC1	I have the resources (i.e., time and money) to support my dog’s heart treatments.	5.70 (1.48)	0.617	0.734/0.835	0.562
	PBC2	I am confident that I can nurse my dog according to the veterinarian’s instructions.	6.16 (1.20)	0.807		
	PBC3	I can be committed to administering prescribed medication (long term) to my dog.	6.32 (1.10)	0.872		
	PBC4	I have the skills to administer oral drugs to my dog.	5.62 (1.64)	0.675		
Subjective Norm ^4^ (SN)	SN1	Family	6.31 (1.43)	0.862	NA	NA
	SN2	Close friend(s)	4.08 (2.11)	0.112		
	SN3	Peers/ Colleagues	3.11 (2.02)	−0.275		
	SN4	Spouse	5.57 (2.18)	0.413		

SD, Standard deviation; Cronbach’s α, Cronbach’s alpha; CR, Composite reliability; AVE, Average Variance Extracted; NA, not applicable for formative construct. ^1^ For reflective constructs the standardized loading is provided; ^2^ For formative construct, the weight of the linear; the combination is given; ^3^ Items INT3 was deleted; ^4^ Formative construct.

**Table 8:** Discriminant validity of the measurement model.

Constructs	(1)	(2)	(3)	(4)	(5)
(1) Attitude	0.741				
(2) Empathic Concern	0.499	0.772			
(3) Intention to Treat	0.610	0.444	0.723		
(4) Perceived Behavioral Control	0.580	0.546	0.636	0.750	
(5) Subjective Norm	0.110	0.151	0.215	0.180	Formative Construct

Diagonals (boldface) represent the square root of the AVE, whereas the other entries represent the correlations.

**Table 9:** Path coefficients of the structural model and results of hypothesis testing (H): The moderating influence of empathic concern (EC) on the relationship between attitude (ATT), subjective norm (SN), perceived behavioral control (PBC), and intention to treat (INT) dogs with heart disease.

Hypothesis (H)	Relationship	Beta	SE	t-Values	*p*-Values	Decision
H1	ATT → INT	0.333	0.082	4.059 *	0.000 **	Supported
H2	SN → INT	0.093	0.050	1.852 *	0.032 **	Supported
H3	PBC → INT	0.363	0.073	5.002 *	0.000 **	Supported
H5	ATT * EMP → INT	−0.033	0.068	0.487	0.313	Not supported
H6	SN * EP → INT	−0.033	0.062	0.530	0.298	Not supported
H7	PBC * EMP → INT	−0.101	0.059	1.718 *	0.043 **	Supported

* t-value > 1.65, ** *p* < 0.05. →: indicates a relationship.

## Data Availability

All individuals included in this section have consented to the acknowledgement.

## Appendix A

### Theory of Perceived Behaviour Questions

1. Intention To Treat
  - I intend to follow up with my dog’s heart treatments.
  - I intend to practice a low salt diet.
  - I intent to stop giving commercial treat(s) to my dog.
  - I intend to administer medications recommended by the veterinarian.
  - I intend to feed my dog with a special formulated diet to support the heart.
2. Perceived Behavioral Control
  - I have the resources (i.e., time and money) to support my dog’s heart treatments.
  - I am confident that I can nurse my dog according to the veterinarian’s instructions.
  - I can be committed to administering prescribed medication (long term) to my dog.
  - I have the skills to administer oral drugs to my dog.
3. Subjective Norm
  - Family.
  - Close friend(s).
  - Peers/ Colleagues.
  - Spouse.
4. Attitude
  - It is wise to bring my dogs for his/her regular check-ups.
  - It is a good idea to ensure that my dog is healthy.
  - It is wise to begin the life-long therapy (medication) for the benefits of my dog’s life.
  - I will update my veterinarian during each check-up on my dog’s condition during treatment at home.
  - I will get in touch immediately with my veterinarian when my dog suddenly looks sick.
5. Empathic Concern
  - I often have tender and concerned feelings for animals.
  - My dog is a family member.
  - I would describe myself as a pretty soft-hearted person.
